# Startle-Induced Epileptic Spasms: A Clinical and Video-EEG Study

**DOI:** 10.3389/fneur.2022.878504

**Published:** 2022-06-15

**Authors:** Zhao Xu, Xianru Jiao, Pan Gong, Yue Niu, Zhixian Yang

**Affiliations:** Department of Pediatrics, Peking University First Hospital, Beijing, China

**Keywords:** startle seizure, epileptic spasms (ES), electroencephalogram, high-frequency oscillations, clinical characteristic

## Abstract

**Objective:**

This study aimed to delineate the detailed characteristics of startle-induced epileptic spasms (ES) and explore the brain regions where startle-induced ES originated.

**Methods:**

Among 581 patients with ES registered in our database, 30 were diagnosed with startle-induced ES according to video-electroencephalogram (EEG) and seizure semiology and were included in this study. Patients' clinical characteristics and ictal high-frequency oscillations (HFOs) were analyzed.

**Results:**

Mean age at the onset of startle-induced ES was 28.1 months. Half of the patients had structural etiology, two of whom were diagnosed with co-existing structural and genetic etiologies. The focal neuroimaging abnormalities were predominant in the frontal cortex (9/15, 60.0%). Fifteen patients (50%) had prominent interictal epileptiform discharges in the frontal and anterior temporal. Ictal HFOs counts of the startle-induced ES in the anterior region were significantly higher than those in the posterior regions (*p* < 0.05). Five patients (16.7%) became seizure-free ≥6 months, and ten (33.3%) showed startle-induced ES cessation ≥6 months. All patients except one had mild to severe psychomotor developmental delay after the onset of seizures.

**Conclusion:**

Patients with startle-induced ES typically had brain lesions and showed drug-resistant. The neuroimaging and EEG findings, including ictal HFOs, support that startle-induced ES often originates from the frontal cortex.

## Introduction

Startle seizures precipitated by sudden and unexpected stimuli were first described by Alajouanine and Gastaut (1). The most common stimulus is auditory, although, in a minority of patients, touch and visual stimuli may also be a trigger (2). Startle seizures were drug-resistant, and the common types were tonic, myoclonic, myoclonic–tonic, and tonic-clonic seizures (3–7). However, only four patients were involved in three studies on epileptic spasms (ES) induced by startle stimuli (8–10). Those patients had startle-induced ES or tonic spasms and spontaneous seizures such as ES, tonic, and focal seizures. Ictal EEG revealed diffuse or focal fast-wave activity (8, 9). In fact, we found that startle-induced ES are not uncommon and easy to ignore in clinical work. Due to few cases have been reported and the clinical features of startle-induced ES are unclear, the major aim of this study, therefore, is to delineate and clarify the characteristics of patients with startle-induced ES.

Patients with startle seizures typically had brain lesions, and the insults occur pre or perinatal, and the focal neuroimaging abnormalities were predominant in the frontal lobes (3, 4, 11). Even in patients without neuroimaging abnormalities, epileptogenic zone resection in the frontal cortex also terminated startle seizures (12, 13). Ictal HFOs on scalp EEG show a strong association with seizure onset zones and may serve as a biomarker for the pathological up states of ES (14–16). Besides, studies have shown that startle seizures are associated with the frontal lobe, specifically the supplementary motor area (12, 17). Are startle-induced ES associated with the frontal lobes similar to other startle seizures? To clarify the origin of startle-induced ES, we applied a fully automatic detection method to characterize the spatial distribution of ictal scalp EEG HFOs (80–150Hz).

## Materials and Methods

### Patients

We retrospectively reviewed 581 patients with ES registered in a database of the Department of Pediatrics, Peking University First Hospital, between January 2012 and January 2021. The seizure type diagnosis of ES were based on the consensus statement of the West Delphi Group (18). We diagnosed WS based on the patient's hypsarrhythmia on EEG at the early presentation of ES and developmental delay. For this study, the inclusion criteria were (1) patients who had ictal video-EEG recordings in our hospital with (2) clear and evident startle-induced ES according to video-EEG, pediatric neurologists' observation, and parents' description. Patients could have multiple types of startle seizures and spontaneous seizures other than ES. Besides, patients with incomplete medical records were excluded. Finally, 30 patients with startle-induced ES were included in our study.

We extracted data from clinical descriptions in the medical records and telephone follow-up with patients. The follow-up period is ≥6 months, with a final evaluation date of December 2021. We investigated clinical data, video-EEG, neuroimaging findings, genetic testing, metabolic examination, the type of seizures, the frequency of seizures, and seizure outcome. According to International League Against Epilepsy (ILAE) criteria, etiology was classified into structural, genetic, metabolic, infectious, immune, and unknown causes (19). Three pediatric neurologists (ZY, ZX, and XJ.) reviewed the records and confirmed seizure types. Epileptic seizure types were categorized according to the Operational Classification of Seizure Types by the ILAE (20). We used the numerical value to describe seizure frequency to maintain consistency in data collection.

### Medication

Adrenocorticotropic hormone (ACTH, <1-year-old, 1IU/kg/d, 14d; >1-year-old, 25 IU/d, 14d) or vigabatrin (VGB) was used to treat West syndrome. For the patients diagnosed with Lennox-Gastaut Syndrome (LGS), methylprednisolone (20mg/kg·d) was used. For other oral antiseizure medication (ASM) such as valproate (VPA), levetiracetam (LEV), topiramate (TPM), lamotrigine (LTG), etc., we follow the medication instructions. During the same period, we only used one drug for treatment to observe the efficacy of a certain drug. At the final follow-up point, compared to baseline seizure frequency, seizure outcomes were classified into three classes: (1) seizure-free, complete elimination of seizures ≥6 months, (2) Startle-induced ES cessation ≥6 months, and (3) non-response, stimuli can still induce ES.

### EEG

Scalp video-EEG monitoring (Nihon Kohden digital video-EEG-1200C with a sampling rate of 500 or 1,000Hz) was performed in all patients with 19 scalp electrodes placed according to the international 10–20 system. Besides, electromyogram (EMG) electrodes were placed in all patients' deltoid muscles and in 3 patients' quadriceps femoris to record myoelectric activity. The recording duration for each patient was 4 hours, and sleep persisted for at least 1 hour. We were aware of the provoking factors of patients by parental description or inadvertently detected during EEG monitoring. Provocative tests such as sound (clap hand), touch (tap the patients' shoulder), and intermittent photic stimulation (IPS) were performed on all patients according to their known provoking factors during EEG recording.

Ictal HFOs (80–150 Hz) were automatically detected using the Delphos detector plugins (Version 1.0.1) within the open-source software Anywave (21, 22). Further data analyses were performed with the MATLAB R2018b (Version 9.5; MathWorks, Natick, MA, USA). We used the longitudinal bipolar montage as a reference and kept the Delphos detector default settings (number of voices 12, vanishing moment 20, threshold type 40, oscillation width threshold 1.4, oscillation frequency spread threshold 10). Based on the default settings (oscillation threshold), the software automatically determines the count of oscillations satisfying the thresholds. We divided the channels into two groups to compare the spatial distribution of HFOs: anterior (Fp1-F3, Fp2-F4, F3-C3, F4-C4, FP1-F7, FP2- F8, F7-T3, F8-T4, Fz-Cz), posterior (C3-P3, C4-P4, P3-O1, P4-O2, T3-T5, T4-T6, T5-O1, T6-O2, Cz-Pz). We recorded the count of HFOs, including 10 s before and 10 s after each startle-induced ES episode. Patients who had ≥3 startle-induced ES during the 4 hours of video-EEG monitoring were included in the analysis.

### Statistics

The SPSS 26.0 software was used for statistical analysis. The Shapiro–Wilk method was adopted to test the normality of measurement data, and Levene's test was used for homogeneity of variance. The paired *t*-test was used to compare the HFOs count between the anterior and posterior regions. A level of *P* < 0.05 was considered statistically significant.

This study was approved by the Ethics Committee of Peking University First Hospital. Written consent was obtained from patients and their parents.

## Results

### Patient Characteristics and Demographics

**Figure 1:**
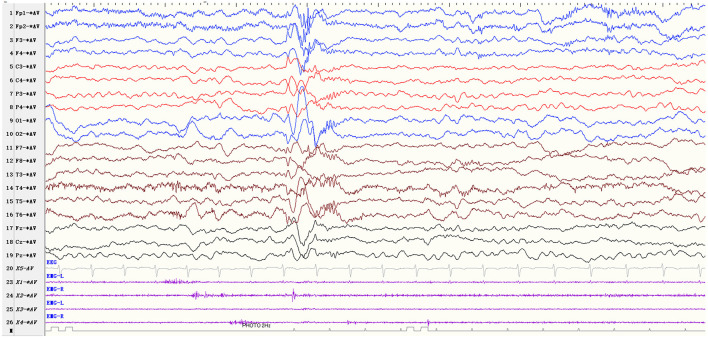
EEG parameters: sensitivity 10 μV/mm, high-frequency filter 70 Hz, low-frequency filter 0.5 Hz Visual stimulus-induced ES (patients 15). The patient showed ES due to the sudden unexpected flash stimulus, and the ictal EEG showed generalized 1–2Hz medium-high amplitude polyphasic slow-waves and 16–20Hz low amplitude fast rhythms.

Based on inclusion and exclusion criteria, we enrolled a total of 30 patients with startle-induced ES. Table 1 summarizes the clinical characteristics of patients (22 males, 73.3%). The mean age at initial seizure presentation and onset of startle-induced ES was 23.1 ± 24.5 months and 28.1 ± 26.5 months, respectively. The mean age of the patients who came to our hospital and were diagnosed with startle-induced ES was 70.7 ± 53.7. Seventeen patients (56.7%) were diagnosed with West syndrome, four of whom subsequently developed into LGS. A total of six patients (20%) were diagnosed with LGS, and 11 patients did not meet the diagnosis of epilepsy syndrome. Startle-induced ES were provoked by sudden and unexpected stimuli. Based on the video-EEG recording, doctor's observation, and parents' description, the provoking stimuli were identified as sound (28, 93.3%), touch (3, 10%), and visual (1, 3.3%). In one patient (patient 15), visual stimulus acting as a startle factor induced ES, which differed from photo-convulsive responses (PCR), and the ictal EEG showed generalized 1–2Hz medium-high amplitude polyphasic slow-waves and 16–20Hz low amplitude fast rhythms (Figure 1). The frequency of startle-induced seizures accounted for more than 50% of all seizures in 12 patients, of whom more than 80% in six patients and one patient had seizures only after sound stimuli. The spontaneous seizures of the other 18 patients were more frequent than startle-induced seizures.

**Table 1:** Demographic and clinical characteristics of patients.

**Characteristic**	**N (%) or**
	**mean ± SD**
Overall	30
Sex, male	22 (73.3%)
Age at initial seizure presentation, months	23.1 ± 24.5
Age at startle seizure onset, months	28.1 ± 26.5
Age at EEG monitoring,^a^	70.7 ± 53.7
Etiology	
Structural	13 (43.3%)
Structural/genetic	2 (6.7%)
Metabolic	1 (3.3%)
Unknown	14 (46.7%)
Epilepsy syndrome	
West syndrome	17 (56.7%)
Lennox-Gastaut syndrome	6 (20.0%)
Provoking stimulus	
Sound	28 (93.3%)
Visual	1 (3.3%)
Touch	3 (10.0%)
Startle-induced seizures accounted for≥80%^b^	6 (20.0%)
Startle-induced seizures accounted for≥50%, <80%^c^	6 (20.0%)
Seizure-free	5 (17.2%)
Startle-induced SE cessation	10 (33.3%)

a *The patients' age when they came to our hospital for EEG monitoring and were diagnosed with startle-induced ES*;

b *The frequency of startle-induced seizures accounted for ≥80% of all seizures*;

c *The frequency of startle-induced seizures accounted for ≥50 and <80% of all seizures*.

### Etiology

The neuroimaging features of patients are presented in Table 2. Based on clinical data, video-EEG, neuroimaging findings, genetic testing, metabolic examination, the patients were diagnosed with structural etiology in 13 patients, structural/genetic etiology in two patients, unknown etiology in 14 patients, and metabolic etiology in 1 patient. One patient with TSC2 gene mutation (c.344G>A) was diagnosed with structural and genetic etiologies. One patient had a *de novo* pathogenic mutation in the GRIN1 gene (c.2365G>A) with bilateral frontal lobe hypoplasia and was diagnosed with structural and genetic etiologies. One patient suffered from methylmalonic acidemia and hydrocephalus.

**Table 2:** Clinical characteristics of patients.

**Etiology**	**No**.	**Neuroimaging**	**Age at EEG monitoring (month)**	**EEG background activity**	**Ictal EEG**	**Interictal EEG**	**Treatment ever tried**	**Startle seizure control**
Structural	1	Periventricular leukomalacia	144	Slow	a b	e	CBZ, VPA, LTG, LEV, OXC	No
	2	Multiple encephalomalacias	120	e	a b	e	VPA, LTG, LEV	Yes
	3	Bilateral frontotemporal cortex and basal ganglia lesions	6	Slow-wave activity	a b	e	VPA, TPM, LEV	No
	4	Left frontal focal cortical dysplasia	71	Slow-wave activity	b c	e	LEV, VPA, TPM, LTG, ZNS, MPS, VNS, KD	No
	5	Bilateral frontoparietal and periventricular white matter lesions	53	Slow	a	e	LEV, VPA, TPM, LTG, KD, MPS	Yes
	6	Periventricular leukomalacia	25	Normal	b	e	VPA, TPM, LEV, OXC,	No
	7	Right parietal focal atrophy	91	Normal	a b c	e	VPA, TPM, CBZ, CZP, LTG, LEV	No
	8	Bilateral frontoparietal and periventricular white matter lesions	132	Slow	a b c	e	VPA, LEV, TPM, CZP	Yes
	9	Bilateral frontal atrophy; bilateral frontal subcortical white matter lesions, periventricular leukomalacia, basal ganglia lesions	17	Normal	a b c	e	ACTH, TPM, OXC	Yes
	10	Left frontal and thalamus lesions	38	Slow-wave activity	a b	e	LEV, TPM, VPA, CBZ, CZP	No
	11	Left parietal and thalamus lesions	180	Normal	a b	e	VPA, CBZ, TPM, LEV, LTG	No
	12	Multiple encephalomalacias	60	Slow-wave activity	a b c	e	LEV, VPA, LTG	No
	13	Hydrocephalus, lateral ventricular drainage, right basal ganglia lesions	48	Slow-wave activity	a b c	e	VPA, LEV, TPM, CZP, LTG	No
Unknown	14	Normal	120	Normal	a b c	e	PB, PHT, CBZ, VPA, LTG, LEV	No
	15	Normal	38	Normal	a b	d	ACTH, VPA, TPM, CZP, LEV, ZNS	No
	16	Bilateral ventricles enlarged	12	d	a b c	d	ACTH, TPM, CZP, VPA, LEV	No
	17	Normal	49	Slow-wave activity	a b	e	VPA, LEV, TPM, LTG, KD	Yes
	18	Delayed myelination, brain atrophy	8	Slow-wave activity	a b	e	ACTH, VPA, LEV, TPM	No
	19	Bilateral frontoparietal demyelination	132	Normal	b c	e	VPA, CBZ, LEV, TPM, LTG	No
	20	Corpus callosum dysplasia	51	Slow	a b	e	VPA, TPM	Yes
	21	Bilateral parietal and left frontal subcortex white matter myelination delay.	31	Slow-wave activity	b	e	LEV, PB, VPA, TPM, ACTH, LTG, VGB	Yes
	22	Normal	30	Normal	a b	e	VPA, CZP, LTG, TPM, LEV, CLB, ACTH	Yes
	23	Normal	26	d	a b c	d	LEV, PB, TPM, ACTH	No
	24	Normal	144	Normal	a b	e	LEV, TPM, CZP, LTG, VPA	No
	25	Normal	132	Slow	a b c	e	LTG, TPM, CZP, KD, MPS, ACTH	No
	26	Normal	72	Normal	a b c	e	VPA, LEV, TPM	No
	27	Brain atrophy corpus callosum dysplasia	8	d	a b	d	ACTH, PB, LEV, TPM, KD, VPA, VGB	No
Structural	28	Frontal lobe dysplasia	9	Slow-wave activity	a b	e	TPM, VPA, VGB	Yes
/genetic	29	Frontal abnormal signal	168	Normal	a c	e	VPA, OXC, LEV, CZP, CBZ, TPM, PB, LTG	No
Metabolic	30	Hydrocephalus	108	Slow	a c	e	LEV, VPA	Yes

*a, generalized 1–2Hz medium-high amplitude polyphasic slow-waves; b, diffuse or focal 10–2 Hz low amplitude fast rhythms; c, diffuse voltage flattening; d, hypsarrhythmia; e, generalized or multiple focal epileptiform discharges; CBZ, carbamazepine; VPA, sodium valproate; LTG, lamotrigine; LEV, levetiracetam; OXC, oxcarbazepine; TPM, topiramate; CZP, clonazepam; KD, ketogenic diet; ZNS, zonisamide; MPS, methylprednisolone; ACTH, adrenocorticotropic hormone; VGB, vigabatrin; CLB, clobazam; PHT, phenytoin; PB, phenobarbital; VNS, vagus nerve stimulation*.

Neuroimaging of patients had diverse and common points. Among the patients with structural etiology (*n* = 15), seven patients were pre-or perinatal asphyxia, four patients had brain injury caused by intracranial infection (three patients due to viral encephalitis and one patient due to tuberculous meningitis), one patient had congenital brain malformation, one patient with brain injury caused by cardiac arrest and hypoxia, and one patient had perinatal hypoglycemia. The patient with TSC2 gene variation had frontal lobe abnormal signals in magnetic resonance imaging (MRI). The focal structural abnormalities were predominant in the frontal cortex in nine out of 15 patients (60.0%).

Among the patients with unknown etiology, the metabolic examination was normal. Nine patients underwent whole-exome sequencing, and no pathogenic gene was found. Neuroimaging was normal in eight patients, and imaging abnormalities were non-specific in six patients and were not considered to be the cause of seizures.

### EEG Finding

Table 2 shows the EEG features of all patients. The interictal EEG mainly showed (1) hypsarrhythmia (4, 13.3%) or (2) generalized or multiple focal epileptiform discharges (26, 86.7%). Patients had epileptiform discharges in waking and sleeping and included at least two of the following wave shape: spike, sharp, spike-wave, sharp-wave, polyspike, and polyspike-waves in paroxysmal or continuous. A common feature of interictal EEG in all patients is massive epileptiform discharges. Fifteen patients (50%) with prominent interictal epileptiform discharges in the frontal and anterior temporal. Background activity showed abnormalities in 18 patients: persistent diffused slow-wave activity in nine patients, slowing (slower frequency than the same age group) in six patients, and hypsarrhythmia in three patients. In one patient, the normal dominant rhythm in the occipital region disappeared and was replaced by massive spikes, spike-wave, sharp, sharp waves complex.

**Figure 2:**
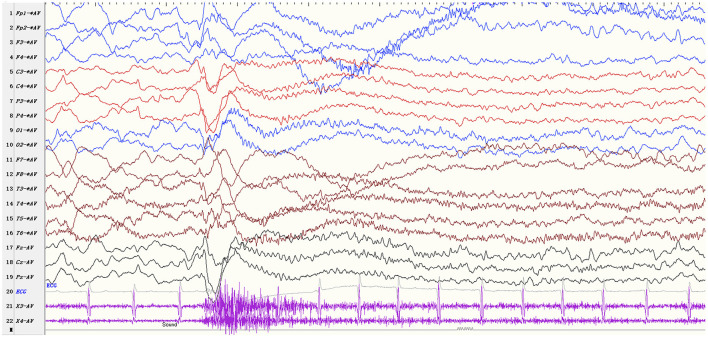
EEG parameters: sensitivity 10 μV/mm, high-frequency filter 70 Hz, low-frequency filter 0.5Hz Startle-induced tonic-spasms (patient 10). The ictal EEG shows generalized medium-high amplitude slow waves followed by diffuse low amplitude fast rhythms and a sustained increase of the EMG activities lasting more than 10 sec.

The ictal EEG manifestations of startle-induced ES were: (1) generalized 1–2Hz medium-high amplitude polyphasic slow-waves (*n* = 26, 86.7%); (2) diffuse or focal 10–20Hz low amplitude fast rhythms (*n* = 27, 90.0%); (3) diffuse voltage flattening (*n* = 14, 46.7%). A single ictal EEG could consist of one part of the above, and the low amplitude fast rhythms could superimpose in the background of voltage attenuation. EMG recording was characterized by rapidly increasing/decreasing muscle activities, often but not always with a diamond-shaped configuration. Patient 10 exhibited ES, followed by a tonic seizure. The ictal EEG showed generalized medium-high amplitude slow waves followed by diffuse low amplitude fast rhythms and a sustained increase of the EMG activities lasting more than 10 seconds (Figure 2).

**Figure 3:**
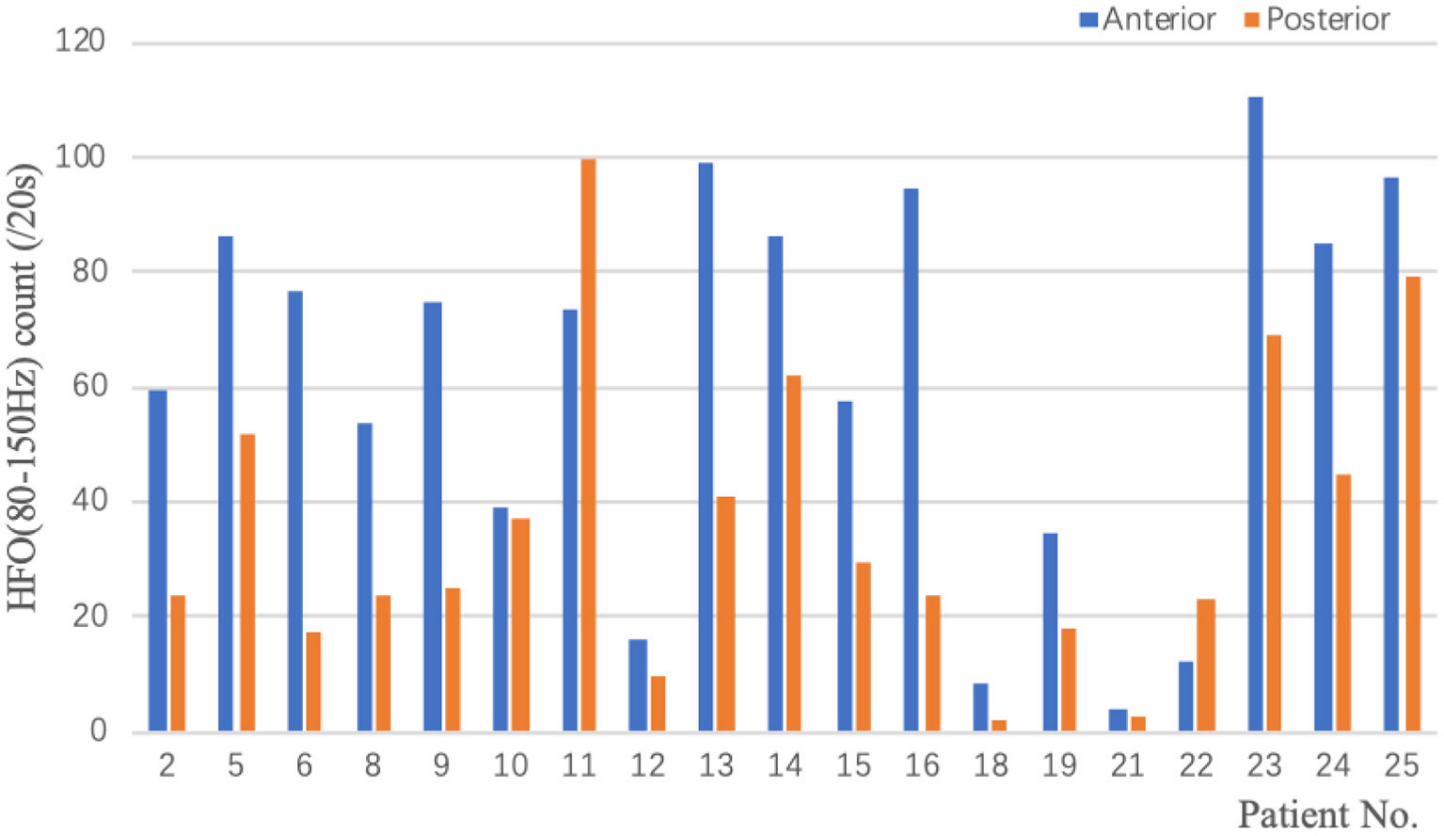
Ictal HFOs of ES on scalp EEG.

A total of 19 patients with 3–7 times startle-induced ES during video-EEG monitoring were included in the analysis of ictal HFOs (Table 3; Figure 3). Eleven patients were not included in the analysis because startle-induced ES was less than three times. As depicted in Table 3 and Figure 3, HFOs of the startle-induced ES in 17 patients typically located in the anterior region corresponding to Fp1-F3, Fp2-F4, F3-C3, F4-C4, FP1-F7, FP2- F8, F7-T3, F8-T4, Fz-Cz. In two patients, HFOs mainly occurred in the posterior region. The two groups of data conformed to the normal distribution (*p* = 0.131 and *p* = 0.112, respectively) and homogeneity of variance (*p* = 0.163). We used the paired *t*-test to compare the HFOs count between the anterior and posterior regions. HFOs counts in the anterior region were significantly higher than in the posterior regions (*p* < 0.05).

**Table 3:** Ictal HFOs of ES on scalp EEG.

**Patient no**.	**Number of startle-induced ES for analysis**	**HFO (80**–**150Hz) count (mean/20s)**	
		**Anterior**	**Posterior**
2	4	59.5	23.5
5	3	86.3	51.6
6	4	76.5	17.3
8	3	54.0	24.0
9	3	75	25.3
10	7	39	37.3
11	6	73.7	100
12	3	16	10
13	4	99.3	41
14	5	86.4	62.2
15	4	57.3	29.5
16	6	94.6	23.5
18	4	8.5	2
19	3	34.6	18
21	4	4	2.5
22	3	12.3	23.3
23	3	110.3	69
24	3	85.3	45
25	4	96.3	79

*Depending on the counts of HFOs from more to less, the color changes from dark blue to white*.

### Seizure Types

The patients' seizures included spontaneous and startle-induced seizures (Table 4). At initial seizures presentation, seizure type on clinical observation was ES in 19 patients (*n* = 1 startle-induced ES and *n* = 18 spontaneous ES), focal motor seizure in eight patients, myoclonic seizure in four patients, tonic seizure in two patients, atypical absence seizure in 1 patient, and tonic-clonic seizure in 1 patient. Five patients had two types of seizures at the onset.

**Table 4:** Seizure characteristics.

**No**.	**Sex**	**Age at initial seizure presentation (months)**	**Types of seizure at onset**	**Spontaneous seizure types**	**Age at startle seizure onset (months)**	**Startle seizure types**	**Provoking stimulus**
1	F	66	Myoclonic	Myoclonic, absence, tonic, FMS	72	ES(I)	Sound
2	F	108	ES	ES, myoclonic	108	ES(I), myoclonic	Sound
3	M	6	ES	ES, myoclonic	6	ES(I)	Sound
4	M	18	ES	ES, atonic, myoclonic, FMS, atypical absence, Tonic	18	ES(I), tonic, myoclonic	Sound
5	M	24	Myoclonic	Myoclonic, atonic, atypical absence, myoclonic-atonic, Tonic	24	ES(I), myoclonic	Sound
6	M	6	ES	ES, tonic-spasms	6	ES(I)	Sound
7	M	12	ES	ES, FMS, myoclonic	84	ES(I)	Sound
8	M	48	ES	ES, atonic	48	ES(I)	Touch
9	M	6	ES	ES, tonic, tonic-spasms	12	ES(I), myoclonic	Sound
10	M	26	ES	ES, tonic-spasms	26	ES(I), tonic-spasms	Sound
11	M	72	ES(startle-induced)	atypical absence	72	ES(I), tonic	Sound
12	M	41	ES	ES, tonic, myoclonic	60	ES(I), myoclonic	Sound
13	F	24	FMS	ES, myoclonic, tonic, FMS	28	ES(I), Tonic	Sound
14	M	36	ES, FMS	ES	36	ES(I), Tonic	Sound, Touch
15	M	24	ES	ES	24	ES(C)	Visual
16	F	1.2	ES, FMS	FMS, ES, atonic	12	ES(I), Tonic, Myoclonic	Sound
17	M	24	Atypical absence	ES, myoclonic, absence	24	ES(C), Myoclonic	Sound
18	M	1	ES, FMS	FMS, ES	6	ES(C)	Sound
19	M	5	ES	ES, absence, tonic, myoclonic	12	ES(I)	Sound
20	F	36	ES	ES, tonic, myoclonic,	36	ES(I), Tonic, Myoclonic	Sound
21	F	6.5	FMS	ES, FMS, myoclonic	14	ES(I)	Sound
22	M	13	ES	ES, atypical absence	13	ES(C)	Sound, touch
23	F	14	Tonic	ES, tonic, myoclonic	14	ES(I)	Sound
24	F	6	FMS	ES	6	ES(I)	Sound
25	M	3	Tonic-clonic, FMS	ES, tonic-clonic, FMS	12	ES(C), tonic	Sound
26	M	36	Myoclonic	—	36	ES(I), myoclonic	Sound
27	M	0.04 (one day)	Myoclonic	Myoclonic, FMS, ES, tonic	2	ES(I), myoclonic	Sound
28	M	5	Tonic	ES, tonic	8	ES(I)	Sound
29	M	13	ES, FMS	ES, FMS	13	ES(I)	Sound
30	M	12	ES	ES	12	ES(I), myoclonic	Sound

*ES, Epileptic spasms; FMS, Focal motor seizure; (I), Isolated; (C), Clusters*.

Patients' spontaneous seizures were diverse, including ES (*n* = 26), myoclonus (*n* = 14), tonic (*n* = 11), focal motor seizures (*n* = 10), atypical absence (*n* = 4), atonic (*n* = 4), absence (*n* = 3), tonic-spasms (*n* = 3), tonic-clonic (*n* = 1), and myoclonic-atonic (*n* = 1). Only five patients showed one type of spontaneous seizure including ES (*n* = 4) and atypical absence (*n* = 1), and one patient never had spontaneous seizures.

According to the video-EEG recordings, sudden unexpected stimuli induced ES in all the patients. Startle stimuli induced only ES in 14 patients (46.7%), and startle stimuli induced other multiple types of seizures in 16 patients (53.3%), including myoclonus (*n* = 11), tonic (*n* = 7), and tonic-spasms (*n* = 1). The characteristics of startle-induced ES were as follows: after sudden stimulation, fast movements of the patients' head, trunk, and limbs occurred simultaneously or independently with varying degrees. The movements were flexor or extensor, or a mixture of extensor and flexor movements, and three patients' movements were asymmetrical. Startle-induced ES showed clusters in five patients (16.7%) and isolated in 25 patients (83.3%).

### Treatment and Outcomes

Tables 2, 5 show the drugs treatment for patients with startle-induced ES. Startle-induced ES often suggested refractory seizures and poorly responded to ASM treatment. Ten patients (33.3%) showed startle-induced ES cessation for ≥6 months. Three patients were treated with vigabatrin (VGB), and two reached startle-induced ES free. Three patients diagnosed as LGS were treated with methylprednisolone, and in one case, startle-induced ES could be controlled. Nine patients still had startle-induced ES after ACTH treatment. The most frequently used drugs were sodiumvalproate (VPA, *n* = 27), levetiracetam (LEV, *n* = 26), topiramate (TPM, *n* = 25), and lamotrigine (LTG, *n* = 16), but the effectiveness was poor. VPA, LEV, TPM, LTG, OXC, and ketogenic diet (KD) ceased one patient's startle-induced ES, respectively. Two patients showed cessation of startle-induced ES after using the clonazepam (CZP). Besides, one patient still had frequent startle-induced ES after applying vagus nerve stimulation (VNS). Five patients (16.7%) became completely seizure-free for ≥6 months after using methylprednisolone, OXC, TPM, CZP, VPA, respectively.

**Table 5:** Treatment efficacy for startle-induced ES *n*(%).

**Treatment**	**Total**	**Non-response**	**Startle-induced ES cessation (≥6 months)**
ACTH	9	9	
MPS	3	2 (66.7%)	1 (33.3%)
VGB	3	1 (33.3%)	2 (66.7%)
TPM	25	24 (96.0%)	1 (4.0%)
VPA	27	26 (96.3%)	1 (3.7%)
LTG	16	15 (93.8%)	1 (6.2%)
LEV	26	26	
CZP	10	8 (80.0%)	2 (20.0%)
PB	5	5	
PHT	1	1	
CBZ	7	7	
ZNS	2	2	
KD	5	4 (80.0%)	1 (20.0%)
OXC	4	3 (75.0%)	1 (25.0%)
CLB	1	1	
VNS	1	1	

*ACTH, adrenocorticotropic hormone; MPS, methylprednisolone; VGB, vigabatrin; TPM, topiramate; VPA, sodium valproate; LTG, lamotrigine; LEV, levetiracetam; CZP, clonazepam; PB, phenobarbital; PHT, phenytoin; CBZ, carbamazepine; ZNS, zonisamide; KD, ketogenic diet; OXC, oxcarbazepine; CLB, clobazam; VNS, vagus nerve stimulation*.

Most patients still had spontaneous seizures (25, 83.3%) and startle-induced seizures (20, 66.7%) after 3–8 kinds of ASM treatment during the long-term follow-up (≥6 months). Two patients were withdrawn from all the drugs by their parents and then showed status epilepticus. Twelve patients (40.0%) had normal cognitive and motor development before the onset of seizures. Twenty-nine patients (96.7%) had mild to severe psychomotor developmental delay after the onset of seizures. But, in one patient with unknown etiology whose seizures were all induced by startle stimulation, his cognitive-motor development remained normal both before and after the onset of seizures. Seven patients (23.3%) could understand simple instructions, identify family and strangers, express basic needs such as hunger, drinking water, and so on.

## Discussion

This study mainly describes the clinical characteristics of patients with startle-induced ES so that clinicians could have a deeper understanding of this startle seizure type. Patients with startle-induced ES specifically have the following features: (1) patients had refractory startle-induced and/or spontaneous seizures and developmental psychomotor impairment; (2) ES were evoked by sudden unexpected sensory stimuli, typically by auditory (93.3%); (3) startle-induced ES originate more in the frontal lobe. Compared to patients with spontaneous ES only, the patients have the same ictal EEG and seizure semiology, while the main differences are that startle-induced ES usually appear later and have a worse response to medication and prognosis (23). Besides, startle-induced ES originate more in the frontal lobe and spontaneous ES may often originate from the posterior parasaggital region (24).

### Clinical Characteristics

There were few reports on startle-induced ES (8–10). A multicenter cohort study of children with post-encephalopathic epilepsy after acute encephalopathy with biphasic seizures and late reduced diffusion showed that two patients had startle-induced ES (8). Saito, Sugai (9) reported that auditory stimuli provoked ES or brief tonic seizures in a patient. Ictal EEG recording showed posterior predominant fast activity and subsequent desynchronization. In addition, interictal EEG showed hypsarrhythmia, and the patient was diagnosed with infantile spasms. Graf (10) also reported a case with spontaneous epilepsy seizures and startle-induced tonic spasms, the latter refractory to all conventional medication except for L-tryptophan. The characteristics of our patients were similar to the patients of previous studies (8–10). At the same time, in our patients, touch and visual stimuli could also induce ES. In addition to ES, our patients have more various types of startle-induced and spontaneous seizures.

Koo and Hwang (25) noted that the localization of focal cerebral lesions affects the age of onset of spasms: occipital lesions were associated with the earliest onset of spasms, and frontal lesions were associated with the latest spasms onset. In our patients, the age at initial seizure presentation and onset of startle-induced ES was late.

### Startle-Induced ES Onset Zone

For neuroimaging, the focal abnormalities were predominant in the frontal cortex (60.0%), and EEG recordings showed that interictal epileptiform discharges were more evident in frontal and anterior temporal regions. Many studies showed that the onset of startle-induced seizures was associated with the frontal cortex (3–5, 17, 26, 27). Nolan et al. (28) noted that the seizure semiology, neuroimaging, and neurophysiological findings supported the involvement of the supplementary motor area in the generation of startle seizures. Besides, our previous study about startle epilepsy in childhood also found that the focal neuroimaging abnormalities were predominantly in the frontal, temporal, and sylvian fissures cortex (7). In the current study, one patient was diagnosed with structural and genetic etiology due to a *de novo* pathogenic mutation in the GRIN1 gene (c.344G>A) and bilateral frontal lobe hypoplasia. More recently, Zhang et al. (29) reported a patient with a *de novo* variant in the GRIN1 gene (c.1595C>A). The clinical phenotype was characterized by developmental encephalopathy, striking stimulus-sensitive myoclonus, and frontal lobe and frontal white matter hypoplasia, similar to our patient. Iwatani et al. (30) reported that ictal HFOs (80–150Hz) of spasms on scalp EEG showed a strong association with neuroimaging abnormalities presumed to be the epileptogenic zone. One study of HFOs (80–250Hz) with long-term scalp EEG monitoring reported that patients with spontaneous ES exhibited significantly higher rates of HFOs in the posterior parasaggital region and significantly lower HFO rates in frontal channels (24). Differently, Job et al. (12) proved that the patients showed a significant increase of HFOs (60–100 Hz) at startle seizures onset over the premotor and prefrontal areas. We also found that ictal scalp EEG HFOs (80–150Hz) of startle-induced ES were predominant in the anterior region, corresponding to our patients' interictal EEG epileptiform discharges (prominent in frontal and anterior temporal regions). Provides evidence that startle-induced ES may originate from the frontal lobe, unlike spontaneous ES.

### Medication

In our study, startle seizures in 25 patients were drug-resistant, with no response to multiple ASM. Previous studies reported that startle seizures were refractory (6, 7), as confirmed by our patients. Five of our patients (17.0%) became completely seizure-free, and ten patients (33.3%) showed startle-induced ES cessation ≥6 months. However, we did not find that a specific drug was particularly effective. Some drugs were frequently used (VPA, LEV, LTG, TPM) in our patients, but the response was poor. Two studies showed that LTG (31) and LEV (32) were effective in startle seizures. But, in the present study, LTG (*n* = 16) and LEV (*n* = 26) were used in our patients, with only one patient responding to LTG. Seventeen patients were diagnosed with West syndrome, nine of whom used ACTH, but they continued to have frequent seizures. One patient showed startle-induced ES cessation after applying methylprednisolone. VGB was only used in three patients diagnosed with West syndrome, and two patients showed a cessation of startle-induced ES. As one of the first-line drugs for treating infantile spasms (33), VGB efficacy for startle-induced ES needs more research to verify.

### Prognosis

Whether seizures were controlled or not, most of our patients (96.7%) still had moderate to severe developmental delay. The outcomes and prognosis of startle-induced ES reported in our study were consistent with the previous studies (8–10). However, one patient in our study had only startle seizures with unknown etiology, and his cognitive-motor was normal before and after the onset of seizures. The patient's clinical features were similar to a case reported by Cokar et al. (34).

### Differential Diagnosis

From this study, we can see that most of the patients were not diagnosed with startle-induced ES for a long time in the past until they came to our hospital for EEG monitoring and were diagnosed with startle-induced ES. By then, most patients have had a long time since their initial startle-induced ES. The main possible reasons for this are: (1) the relationship between ES and startle stimuli are not noticed by doctors during EEG monitoring; (2) ES are mild and difficult for parents and doctors to detect; and (3) startle-induced ES are easily confused with other startle reflex and fail to be diagnosed (35). These reasons may lead to misjudgment of the patient's condition. Here, we provide some differentiation of startle-induced ES from other startle reflexes: (1) severe developmental delay is present in most patients; (2) patients are often accompanied by other spontaneous seizures; (3) interictal EEG is significantly abnormal; and (4) the relationship between provoking factors and seizures can be clarified on the ictal EEG, EMG, and video recordings of seizures.

## Conclusions

In summary, patients with startle-induced ES typically had brain lesions. Neuroimaging and EEG findings, including ictal HFOs, reveal that startle-induced ES is related to the frontal cortex, whether there is a focal abnormality in neuroimaging or not. Besides, startle-induced ES are intractable seizures, accompanied by various types of startle-induced and/or spontaneous seizures. Most of the patients have mild to severe psychomotor developmental delay.

## Data Availability Statement

The original contributions presented in the study are included in the article/supplementary files, further inquiries can be directed to the corresponding author.

## Ethics Statement

This study was approved by the Ethics Committee of Peking University First Hospital. Written consent was obtained from patients and their parents. Written informed consent to participate in this study was provided by the participants' legal guardian/next of kin. Written informed consent was obtained from the minor(s)' legal guardian/next of kin for the publication of any potentially identifiable images or data included in this article.

## Author Contributions

All authors listed have made a substantial, direct, and intellectual contribution to the work and approved it for publication.

## Conflict of Interest

The authors declare that the research was conducted in the absence of any commercial or financial relationships that could be construed as a potential conflict of interest.

## Publisher's Note

All claims expressed in this article are solely those of the authors and do not necessarily represent those of their affiliated organizations, or those of the publisher, the editors and the reviewers. Any product that may be evaluated in this article, or claim that may be made by its manufacturer, is not guaranteed or endorsed by the publisher.
